# Carbapenem-Resistant *Pseudomonas aeruginosa’s* Resistome: Pan-Genomic Plasticity, the Impact of Transposable Elements and Jumping Genes

**DOI:** 10.3390/antibiotics14040353

**Published:** 2025-03-31

**Authors:** Theodoros Karampatakis, Katerina Tsergouli, Payam Behzadi

**Affiliations:** 1 Department of Clinical Microbiology, University Hospital Kerry, V92 NX94 Tralee, Ireland; theodoros.karampatakis@hse.ie (T.K.); aikaterini.tsergouli@hse.ie (K.T.); 2 Department of Microbiology, Shahr-e-Qods Branch, Islamic Azad University, Tehran 37541-374, Iran

**Keywords:** *Pseudomonas aeruginosa*, genome, plasticity, antimicrobial resistance, transposable elements, mobile genetic elements, jumping genes

## Abstract

*Pseudomonas aeruginosa*, a Gram-negative, motile bacterium, may cause significant infections in both community and hospital settings, leading to substantial morbidity and mortality. This opportunistic pathogen can thrive in various environments, making it a public health concern worldwide. *P. aeruginosa*’s genomic pool is highly dynamic and diverse, with a pan-genome size ranging from 5.5 to 7.76 Mbp. This versatility arises from its ability to acquire genes through horizontal gene transfer (HGT) via different genetic elements (GEs), such as mobile genetic elements (MGEs). These MGEs, collectively known as the mobilome, facilitate the spread of genes encoding resistance to antimicrobials (ARGs), resistance to heavy metals (HMRGs), virulence (VGs), and metabolic functions (MGs). Of particular concern are the acquired carbapenemase genes (ACGs) and other β-lactamase genes, such as classes A, B [metallo-β-lactamases (MBLs)], and D carbapenemases, which can lead to increased antimicrobial resistance. This review emphasizes the importance of the mobilome in understanding antimicrobial resistance in *P. aeruginosa*.

## 1. Introduction

As of 22 March 2025, the List of Prokaryotic names with Standing in Nomenclature (LPSN) (https://lpsn.dsmz.de/, accessed on 22 March 2025) reports 352 validly published *Pseudomonas* species with correct names (child taxa) (https://lpsn.dsmz.de/genus/pseudomonas, accessed on 22 March 2025). *Pseudomonas aeruginosa* is a motile Gram-negative opportunistic human pathogen which contributes to acute-/chronic- and community-acquired-(CAIs)/hospital-acquired infections (HAIs) with high rates of morbidity and mortality [1,2,3]. *P. aeruginosa* belongs to non-fermenting Gram-negative bacteria (NFGNB) and therefore, it is not able to ferment sugars to provide needed energy for its vital activities [1,2,4]. Moreover, *P. aeruginosa* belongs to the ESKAPE group composed of significant pathogens, e.g., *Enterococcus faecium*, *Staphylococcus aureus*, *Klebsiella pneumoniae*, *Acinetobacter baumannii*, *P. aeruginosa* and *Enterobacter* spp. Recently, the ESKAPE group has been extended to ESKAPEE with *Escherichia coli* as a new member. The members of this known pathogenic group are noted for their contribution in the global enhancement and dissemination of antimicrobial resistance (AMR) phenomena. The AMR strains of these pathogens are named as “superbugs” [5,6,7,8,9]. Indeed, the multi-drug resistant (MDR) strains of *P. aeruginosa* or *P. aeruginosa* superbugs have been noted as a life-threatening global concern via different health organizations, including the World Health Organization (WHO), UK Public Health England, and the US Centers for Disease Control and Prevention (CDC) [10]. Moreover, according to the 2024 WHO Bacterial Priority Pathogens List (WHO BPPL), 24 pathogens (15 families of antimicrobial-resistant bacterial pathogens, including *P. aeruginosa* superbugs) have been updated, which underscores their global impact in terms of burden. Based on recent reports of the global reduction of Carbapenem-resistant *P. aeruginosa* (CRPA) infections, the 2024 WHO BPPL has reclassified and downgraded CRPA infections from a critical to a high priority level [11]. *P. aeruginosa* is recognized as an armed pathogen with a magnitude and strong arsenal such as virulence factors versatility (a powerful virulome), acquired and inherent resistance versatility (a powerful resistome), and metabolic versatility (a powerful metabolome). Therefore, *P. aeruginosa* develops high resistance to a wide range of antibiotics and disinfectants, and simultaneously is highly adaptable, with different environmental factors, extreme habitats, different carbon resources (to capture energy), and different temperatures up to 42 degrees Celsius [12,13,14]. Due to its remarkable ability to thrive in diverse environments, *P. aeruginosa* poses a significant global concern for public health care centers and systems [1,2].

*P. aeruginosa*, an effective pathogenic agent of HAIs, is known as a common cause of bacteremia, respiratory tract infections [e.g., pneumoniae, cystic fibrosis (CF)] in, urinary tract infections (UTIs) and catheter-associated UTIs (cAUTIs), wound infections (e.g., surgical site and burn wound infections) [15,16,17,18,19,20,21]. The immunocompromised patients in hospitals, particularly in intensive care units (ICUs), are the major target hosts for *P. aeruginosa*. Hence, *P. aeruginosa* is responsible for severe and chronic infections with high rates of morbidity and mortality, which is the outcome of biofilm formation, the production of alginate, and the appearance of mucoid phenotypes [9,15,22]. The occurrence of biofilm-associated infections may result from: (i) untreated infections, (ii) inappropriate/indefinite treatment of infections, and (iii) the employment of medical devices (e.g., implants, prosthesis, and catheters) in patients. The indefinite treatment may be related to the treatment strategies. Recruitment of ineffective pharmaceutical therapies, the appearance of antimicrobial resistance features, and the occurrence of genetic exchanges such as horizontal gene transfer (HGT) are the main reasons for indefinite/inappropriate treatments [2,4,23,24,25,26,27]. Today, the concern of microbial biofilm formation and biofilm-related antimicrobial resistance features not only involves the clinical settings, but also the environmental settings. In this regard, soil and wastewater are identified as the main environmental reservoirs that contribute to the development and dissemination of AMR genes (ARGs) [23]. The spread of ARGs can be easily accomplished through a wide range of genetic elements (GEs) including jumping genes and mobile genetic elements (MGEs) like genomic islands (GIs), plasmids, transposons (Tns), integrons (Ints), insertion sequences (ISs), etc. via the HGT phenomenon [23,28,29]. Now, it is known that the HGT phenomenon mostly occurs in microbial biofilms or microbial mats. On one hand, around 80% of bacterial populations are found in different types of microbial mats at different types of interfaces including air–air (bubble biofilm), liquid–gas, liquid–liquid, solid–liquid and solid–gas [23,30,31,32]; and on the other hand, the bacterial biofilms in different environments such as pharmaceutic-industrial wastewater, domestic wastewater, agricultural soil and wastewater and hospital wastewater) have been detected as effective hotspots (hs) for ARGs dissemination among bacterial pathogens [23,33,34,35,36]. So, the ARGs are able to be disseminated in air, soil, water, animals, and humans via antimicrobial-resistant strains of different bacterial pathogens like *P. aeruginosa* [35]. Because the global concerns relating to AMR feature among bacterial pathogens, e.g., CRPA strains and the ineffective antimicrobial treatment strategy of infections caused by CRPA in clinical and public healthcare centers and systems around the world, the current literature review strives to illustrate the importance of GEs in the development and dissemination of AMR feature in *P. aeruginosa*.

## 2. Genomic Pool, Pan-Genome and Genomic Plasticity

A bacterial genome, such as that of *P. aeruginosa*, comprises both conserved and variable genetic regions [37,38]. Maintaining a stable genome is crucial for the survival of all living cells, including bacteria. However, bacterial cells also require genomic instability and plasticity at various levels to adapt to changing environments [5,37]. In other words, a higher degree of genomic plasticity allows for greater adaptability and flexibility in response to environmental changes [5].

A set or a pool of genes that contribute to a determined bacterial species is known as a pan-genome [5,39,40,41]. A pan-genome consists of three genomic sections, including core (persistent) genome [which is shared among all (at least 95%) strains of a bacterial species], shell (accessory/dispensable/flexible/adaptive) genome (which is shared among at least 15% and less than 95% of the strains in association with a bacterial species), and cloud (strain-specific/unique/singleton) genes which participate in less than 15% of the genomic strains in association with a bacterial species [9,42]. Indeed, the core genome is composed of the essential genes, e.g., house-keeping genes, which are involved in the cells’ daily and vital activities [5,43,44].

According to the National Center for Biotechnology Information (NCBI) Datasets, the genome of the PAO1 strain of *P. aeruginosa* (Genome assembly ASM676v1/ReSeq:GCF_000006765.1/ (https://www.ncbi.nlm.nih.gov/datasets/genome/GCF_000006765.1/, accessed on 22 March 2025)) is known as the reference genome. According to this dataset, *P. aeruginosa* PAO1 encompasses a genome size of 6.3 Mb [containing 5697 genes and 5572 coding sequences (CDS) or open reading frames (ORFs) with a GC percent of 66.5% (https://www.ncbi.nlm.nih.gov/datasets/genome/GCF_000006765.1/) accessed on 22 March 2025].

As previous reports show, there is a limited number of bacterial species like *P. aeruginosa* that encompass a highly adaptable genome. The pan-genomic size of *P. aeruginosa* varies from 5.5 to 7.76 Mbp, which depicts a high plasticity of the genomic pool with a high versatility of genes [10,45]. *P. aeruginosa* possesses a remarkably large genome with brilliant genetic and functional versatility with a considerably high percentage of regulatory genes. In contrast to *P. aeruginosa*, large bacterial genomes in different bacterial genera are mostly composed of gene duplications [46].

The results obtained from previous studies have revealed that the chromosomal (core/persistent genome) GC content of *P. aeruginosa* is 65–67% [28], while the flexible genome of *P. aeruginosa* typically has a lower GC content than that of its core genome [13]. As Table 1 shows, the GC content of PAGIs (as a part of the accessory genome) varies from 50.5 to 66.1%. However, it should be noticed that genomic pressures acting on the *P. aeruginosa* pan-genome eventually eliminate the GC content differences between core and accessory genomes, over time [13]. It is possible to observe a substantial number of conserved genes across all *P. aeruginosa* strains, alongside a diverse array of accessory genes that vary between the same bacterial strains. The accessory genome supports the bacterial adaptability in different environmental niches [10,45]. The content of accessory genome reveals that the main resource of this portion of genomic pool of *P. aeruginosa* is acquired via HGT [47].

Now it is understood that the integration of accessory genes into the *P. aeruginosa* core genome is not a random process. Instead, these genes tend to cluster in specific regions, known as regions of genomic plasticity (RGPs) [63]. The RGPs consist of two major GEs, including genomic islets (GILs) with genetic sequences less than 10 kb and GIs with genetic sequences more than 10 kb [64,65,66,67]. To date, more than 100 RGPs have been detected throughout the *P. aeruginosa* chromosome [47,60]. The RGPs and other certain loci within the bacterial core genome have been recognized as the location where the insertion of acquired genes takes place. In this regard, the *tRNA* genes within the core genome are effective targets for insertion of different types of GEs (e.g., GIs). The GIs in *P. aeruginosa* are named as PAGI [28,66,68]. In a study performed by Poulsen et al. [69], the results showed that the essential core genes constitute 6.6% of the *P. aeruginosa* genome. Furthermore, 93.4% of the *P. aeruginosa* genome is covered by regions of genomic plasticity, which are known as strain-specific fragments [10,63,69]. The results obtained from previous studies indicate that acquired genes from ARGs and heavy metal resistance genes (HMRGs) to virulence genes (VGs) and metabolic genes (MGs) can be transferred to *P. aeruginosa* strains via different types of GEs, such as MGEs through the HGT [45,70,71]. These MGEs may build a huge arsenal for bacterial strains of the *P. aeruginosa* to be armed with a wide range of weapons. This property makes them capable of adapting to different ecological niches via different mechanisms and strategies [45,70,71]. While *P. aeruginosa* exhibits significant genomic plasticity, it remains uncertain whether its pan-genome is considered open or closed [28,72]. Recognizing the crucial role that various GEs play in the plasticity of the *P. aeruginosa* genomic pool, we will discuss several key GEs in the following sections [37].

## 3. Horizontal Gene Transfer (HGT) Mechanisms and the Role of Genetic Elements (GEs)

In general, two types of AMR features, including intrinsic and acquired resistance, have been detected. The acquired resistance feature is a highly rated phenomenon within natural environments like bacterial biofilms and mats. Furthermore, HGT, vertical gene transfer (VGT), and DNA mutations are considerable phenomena relating to the dissemination and development of AMR features among a wide range of bacterial strains, such as *P. aeruginosa* [26,73]. The HGT of ARGs is generally achieved by the MGEs (such as integrative and conjugative elements (ICEs), Ints, Tns, and plasmids), where the ARGs are located for the most part [74]. Moreover, the HGT of ARGs can be increased via a wide range of different factors, including nanomaterials, temperature, pollutants (e.g., non-antibiotic drugs), antibacterial agents, natural factors (e.g., CO_2_), etc. [73,75,76,77,78,79,80]. As aforementioned, dissemination and propagation of ARGs can be mediated by different types of GEs, because GEs act as effective vehicles, in this regard. These vehicles (GEs) are transmitted via four different mechanisms of HGT, including transformation (extracellular naked DNA molecules uptake), transduction [virus-(bacteriophage (Bφ))-mediated DNA transfer], conjugation (cell-to-cell direct contact DNA transfer) and vesiduction [vesicle-related GEs (e.g., DNA) transfer from a donor bacterial cell to a recipient bacterial cell] [23,81,82,83].

### 3.1. Transformation

Transformation is a type of HGT mechanism in which naked DNA gets imported into the recipient bacterial cell. These cell-free or extracellular DNAs (eDNAs) are ubiquitous and can be released from both damaged cells and dead cells [73,84,85]. The transformation mechanism is sensitive to nucleases like DNases and other materials that may lead to eDNA degradation. This feature has been recognized as a disadvantage of the transformation mechanism in comparison with other HGT mechanisms like conjugation [73]. According to previous studies, >80 bacterial species, including *P. aeruginosa*, are able to recruit transformation mechanisms for the acquisition of different genes, e.g., ARGs, etc. [73,86,87]. As we know, clinical and environmental settings act as vast reservoirs of genetic materials like eDNAs [85]. In this regard, type IV pili (T4P) contribute to natural transformation. The ComEA DNA translocation machinery, which is located in the bacterial periplasm, receives the eDNA [88,89]. The retracted eDNA can be inserted into a part of the bacterial genome via transposition or recombination. In the case of plasmids, they can be kept as independent replicons within the bacterial cytoplasm [90]. As aforementioned, plasmids play a significant role in the accessory genome due to their capacity to carry diverse arrays of genes from ARGs and VGs to MGs [5,13,23]. In accordance with recorded reports, *P. aeruginosa* is able to express an abundant amount of eDNA in the process of biofilm expansion mediated by twitching motility, within biofilms and in static broth cultures [91,92,93]. The single-stranded DNAs (ssDNAs) are transferred across the bacterial membrane via the type II-related secretion system and T4P [94,95]. In a project performed by Nolan et al. [87], the investigators showed that the transformation mechanism can occur in *P. aeruginosa* strains. In other words, *P. aeruginosa* strains are able to acquire different types of genes in both forms of plasmids and chromosomal DNA under different conditions. It seems that the occurrence of the transformation mechanism can be enhanced among bacterial cells of *P. aeruginosa* when the expression of T4P increases [87]. Perhaps the transformation mechanism is an effective approach for the acquisition of ARGs and VGs in *P. aeruginosa* strains. Hence, this mechanism may be playing a significant role in the rise of MDR strains of *P. aeruginosa* worldwide [87].

### 3.2. Conjugation

It seems that conjugation (also known as bacterial sex) is the predominant mechanism among the four mechanisms of HGT, and in particular regarding the plasmid transfer. The conjugation mechanism occurs via direct cell-to-cell connection, between the bacterial cells from a single genus or different genera, in which the ARGs, HMRGs, VGs, MGs etc., can be transmitted from a donor bacterial cell to the recipient [73,74,80,96,97]. The transmission of DNA molecule(s) from the donor to the recipient bacterial cell is dependent on retraction of the pil-T-regulated trichome along with the DNA uptake [73,98]. Indeed, pili are multi-task tubular appendages that are involved in adhesion, conjugation, and biofilm formation [98]. As previous studies show, Gram-negative bacteria like *P. aeruginosa* encompass a quasi-permeable membrane. This property makes the Gram-negative bacterial membrane conductive to movement and supports an easy transmembrane transfer of plasmids between a donor bacterial cell and the recipient [77]. Thus, the transmission of plasmids, including those harboring ARGs, is preferentially occurring among Gram-negative bacteria, e.g., *P. aeruginosa*, via the HGT of conjugation [73]. In conjugation, like other HGT mechanisms, different types of MGEs, including GIs [e.g., chromosomal islands, pathogenicity islands (PAIs), resistance islands (REIs)], ICEs, Ints, and plasmids have an influential role in ARGs dissemination [99,100,101,102]. In contrast to non-conjugative plasmids (or mobilizable plasmids that are not capable of encoding the complete conjugation machinery), the conjugative plasmids bear type IV secretion system (T4SS) genes [103]. ICEs are recognized as a part of bacterial chromosomes with no specific characteristics. They involve those MGEs that are capable of encoding genes which are related to their own conjugation, excision, and integration. Therefore, these GEs can be transmitted to other bacterial cells via both VGT and HGT. The integrative mobilizable elements (IMEs), as another part of MGEs, are capable of encoding genes that are related to their own excision and integration. As the IMEs suffer from the lack of conjugative transmission-related genes, they should hijack the conjugative apparatus of ICEs. The IMEs have a pivotal role in the spread of ARGs via harboring them [104,105,106,107,108]. Furthermore, megaplasmids are known as a treasure trove for a diversity of accessory genes, including ARGs [e.g., acquired carbapenemase genes (ACGs)], conjugation, maintenance, plasmid replication, etc. [109,110]. In this regard, the incompatibility group P-2 (IncP-2) megaplasmid is a flexible vehicle harboring cassette genes of CRGs in class 1 Int/Tn [110].

The bacterial replicons are categorized into two major groups: chromosomes and secondary replicons. The largest replicon bearing the highest number of core genes is known as chromosome, while secondary replicons are classified into four groups: second chromosome, chromid, plasmid, and megaplasmid [111]. However, it has been recommended not to use the term “second chromosome” and instead, the term “chromid” is more suitable [111,112]. Indeed, plasmids and megaplasmids are replicons that bear acquired genes, often through the HGT phenomenon. Moreover, as previously mentioned, the GC content (known as an effective genomic signature) of these replicons is significantly lower than that of the chromosome as the core genome [111,112]. Despite the absence of any rigorous classification for distinguishing plasmids and megaplasmids, the size of the replicons determines their categories. DiCenzo and Finan suggested that those secondary replicons with a size of <350 kbp are known as plasmids, while those with a size of >350 kbp are recognized as megaplasmids. This criterion is based on 10% of the median size of the bacterial genome. Thus, the terms “plasmid” and “megaplasmid” can be varied based on the bacterial genus [111].

Plasmids, based on DNA transfer-related protein machinery (including relaxase, type IV coupling protein (T4CP), and T4SSs), are classified into three groups: conjugative, mobilizable (MOB), and immobilizable ones. The mobile plasmids have a considerable role in the dissemination of AGRs in infectious diseases, epidemics, and outbreaks [113].

“Chromid” is a term composed of ‘chro’ (derived from ***chro***mosome) and ‘mid’ (derived from plas***mid***). This replicon depicts the intermediate replicons between chromosomes and plasmids. Chromids possess similar replication systems to plasmids and megaplasmids. In contrast to plasmids and megaplasmids, chromids harbor at least one core gene (e.g., a house-keeping gene involved in a cell’s vital activity). The average and median size of chromids are ~two times more than that of megaplasmids [111,112]. It seems that the mobility and successful transmission of megaplasmids are lower than that of plasmids. Although both megaplasmids and chromids act as conjugative machinery, the former can be transferred between bacterial cells in nature, while the latter cannot be transferred via HGT in nature (it has not been observed) [111,112]. All in all, the rate and frequency of acquired genes in megaplasmids and chromids are lower than that of plasmids.

### 3.3. Transduction

A phage-mediated transduction is accomplished by phage attachment to the bacterial cell host and viral DNA injection into the bacterial cell host. Then, a lysogenic phage (known as a temperate phage) does integrate its own genome into the bacterial host cell’s chromosome to make a prophage in the first step and then can continue as a lytic phage through the lytic cycle process [23,114]. A lytic phage, by hijacking its host cell’s genome replication machinery, proliferates right after its own DNA injection into the bacterial host cell’s DNA. When the lytic cycle is completed, the bacterial DNA may have two different destinies, including integration into a new bacterial host cell’s chromosome via homologous recombination or appearing as an autonomous replicon like a plasmid [23,114,115]. Moreover, the viral genome fragments are able to infect new bacterial host cells. Those phages, which are capable of infecting a wide range of bacterial cells in different taxonomic classifications of orders, may raise a serious concern regarding the spread of different types of genes [116,117,118]. The ARGs can be transferred to bacterial host cells via Bφs. Both prophages and Bφs are capable of encoding ARGs [115,119]. As we know, the spread of viruses and subsequently their metagenome (virome) in different environments, including clinical (terrestrial) and aquatic samples, are significantly high. However, the dissemination of phage-mediated ARGs has been neglected for the most part [115,118].

Moreover, because of the abundance of Bφs, the transduction can occur anywhere, through three different mechanisms of generalized transduction [mispackaging of bacterial DNA fragments from *pac* site homologs (e.g., chromosomal DNA or plasmid DNA) but not phage DNA into capsids at random], lateral transduction (in which large segments of bacterial genomes are transferred by temperate phages) and specialized transduction [it is accomplished only by temperate phages like phage lambda; viral encapsidation of the host cell genes (the segment in adjacent to the *att* site of the prophage) and prophage genome; successful completed specialized transduction can be observed 1/100 transducing particles] [73,120,121,122,123,124,125,126]. Those phages (like phage lambda) that recruit a specialized transduction mechanism have their genomes integrated into the genome of their own bacterial host cells, between the operon genes of galactose metabolism and biotin biosynthesis. Thus, these MGs are capable of being transferred via the HGT phenomenon [125].

It seems that the specialized transduction mechanism has considerable involvement in ARGs transmission. The frequency of the generalized transduction mechanism is significantly low, and the host DNAs have been detected in 1–6% of the virions [124,125,127,128]. The lateral transduction is involved in HGT of VGs, PAIs, ARGs, MGs, etc. [125,129]. In the transduction mechanism, the acquired genes, including ARGs, are packaged within DNA platforms and can be easily transferred into infected bacterial cells [120].

### 3.4. Vesiduction

The vesiduction mechanism, as the fourth mechanism of HGT, is mediated by the outer-membrane vesicles (OMVs), which act as GEs vectors and vehicles [23,130]. The first three HGT mechanisms can be accomplished via MGEs, including Ints, plasmids, and Tns. Vesiduction, as a newly recognized mechanism of HGT, is involved in the transmission of different GEs, such as both groups of nucleic acids involving DNAs (e.g., viral DNA, chromosomal DNA and plasmids) and RNAs (comprising mRNA, tRNA, rRNA and sRNA), proteins, lipids, etc. [82,131]. In an investigation conducted by Dell’Annunziata et al. [132], plasmids containing resistance genes were recruited. They showed that OMVs in *K. pneumoniae* act as effective vectors for the dissemination of ARGs among different microbial populations such as *P. aeruginosa*. Thus, the OMVs as 50–250 nm-diameter vesicles made of Gram-negative bacterial outer membrane are influential vectors of different types of GEs, e.g., plasmids harboring β-lactam resistance genes [132]. These OMVs contribute to HGT mechanisms and have a pivotal role in different types of interactions involving bacterial cells–bacterial cells, bacterial cells–environmental factors, and bacterial cells–hosts [23,133]. Due to this knowledge, the vesiduction mechanism, as the fourth mechanism of HGT, has been detected as an effective procedure in association with the rapid dissemination of ARGs via the transmission of plasmids among bacterial populations such as *P. aeruginosa* [82,134].

## 4. Genetic Elements (GEs) and Their General Characteristics

The presence of some short repetitive sequences (called microsatellites) within the bacterial genome leads to genomic interspersion. Microsatellites are short (1–6 bp) repetitive segments of DNA that can occur throughout the bacterial genome. Microsatellites are weak and unstable regions because they are susceptible to a wide range of genetic variations, including mutations, deletions, duplications, and insertions. The frequency of microsatellites in eukaryotic genomes is high while the frequency of these short/small tandem repeats (STRs) in bacteria is limited and very low [37,135,136]. Unstable genomic regions like microsatellites, which are known as STRs, are highly mutable and directly contribute to gene family expansion and evolution [37,137]. Moreover, the STRs may be involved in ARGs acquisition via transposons (Tns) in AMR strains of *P. aeruginosa* [138]. Tns (or transposable elements (TEs)), which are known as jumping genes, together with other TEs and MGEs or mobilome, are continuously involved in flux and altering the genomic composition of the living cells, e.g., bacterial pathogens of *P. aeruginosa* [139].

MGEs or mobilome involves a wide range of movable GEs, e.g., bacterial interspersed mosaic elements (BIMEs), GIs, ISs, inteins, Ints, introns (MITEs), plasmids, repetitive extragenic palindromes (REPs), retroelements, Tns, and transposable Bφs (prophages) [37]. Mobilome is categorized into two main groups of intercellular MGEs (e.g., plasmids and prophages, which move from a bacterial cell to another) and intracellular MGEs (all GEs that cannot be transferred by themselves but are moved through an integration into plasmids or bacteriophages/TEs) [44]. TEs are discrete DNA segments that are ubiquitous and capable of both replication and/or transmission. TEs can be classified into two groups: conservative TEs, which may lead to insertion only, and replicative TEs, which may lead to replication and cointegrate appearance. So, the main characteristics of TEs are duplications, genomic rearrangements, and mutations [37,140,141]. A TE is identifiable via its basic structure containing a transposase (*tnp*) gene (encoding Tnp enzyme, which is responsible for mobilization of the element) surrounded by a pair of terminal inverted repeats (TIRs) [139]. Furthermore, intracellular MGEs or TEs involve MITEs, Tns, and ISs, which bear GEs like Ints and introns [44,139].

Both ISs and Tns can be located on either plasmids or chromosomes [142]. The occurrence of a wide range of deletions and rearrangements leads to the disruption of MGEs-related GIs mobility. Due to this fact, the transmission mechanisms of a major group of MGEs cannot be identified accurately [63]. Interestingly, GIs as a subclass of MGEs are independent and discrete DNA segments with three forms of mobility [mobile (e.g., ICEs), immobile (e.g., microsatellites), and those that previously were mobile but not anymore (e.g., IMEs, conjugative Tns and some prophages)]. GIs have a remarkable role in genome plasticity, evolution, and bacterial adaptation [29,66,143,144]. MGEs and in particular, ISs, Tns, Ints, plasmids, toxin/antitoxin systems (TAS) and GIs have a pivotal role in the dissemination of a wide range of ARGs like ACGs [ACGs/β-lactamase genes including classes A, B (metallo-β-lactamases (MBLs)], and D carbapenemases. MBLs are highly noted in CRPA [28,145,146,147]. These broad genetic facilities build the *P. aeruginosa* accessory genome and play an essential role in the dissemination of ARGs (like ACGs) and progressive spread among bacterial pathogens, e.g., *P. aeruginosa* [13].

## 5. Insertion Sequences (ISs), Integrons (Ints), Transposons (Tns), Plasmids and Toxin–Antitoxin Systems (TASs)

**Figure 1:**
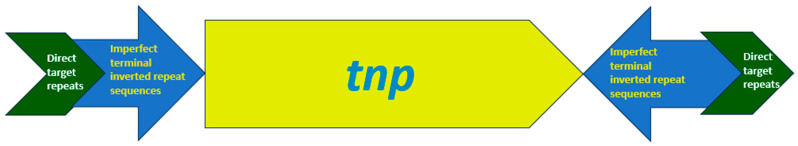
A schematic structure of insertion sequence *6* (IS*6*) as a portion of the accessory genome in detail. One or two transposases (Tnps) as the core structure flanked by two conserved sequences of IRs on the left and right sides. The insertion of ISs occurs on the target DNA via the appearance of short DRs.

ISs have an important role in bacterial genomic plasticity. ISs possess TIR and this characteristic enables them to contribute to independent transposition or duplication in where they are situated (Figure 1) [37,148]. It should be mentioned that those TEs like Tns with high copy numbers within genomes, bearing short conserved TIRs, are known as MITEs. Normally, a prokaryotic MITE is known by its typical architecture including an AT-rich internal sequence (less than 500 bp for the most), a TIR pair with a length of ~4 to 30 bp, and a pair of flanking direct repeats (DRs). Because of MITEs’ specific structures, their integration tendency into AT-rich intergenic regions of target DNA has been recognized [149,150].

The prevalence of MITEs in prokaryotes (bacteria) is largely attributed to the insertion of ISs [44,139,151,152] because ISs are the most abundant autonomous TEs in these organisms, and they can facilitate the movement of associated MITEs *in trans* configuration [44,139,151,152]. MITEs, like other Tns, employ a cut-and-paste movement strategy and mechanism [153,154,155,156].

The MITEs were detected in *Neisseria gonorrhoea* and *N. meningitidis* for the first time. However, the presence of MITEs has been detected in different bacterial groups, including *Enterobacterales* and *Pseudomonads* [139].

Totally, the transposition of TEs is an autonomous feature supported by the related *orf* (*tnp*) genes which can be achieved through two main routes including recruitment of RNA (class 1 TEs/retrotransposons), and employment of DNA (class 2 TEs/DNA Tns) platforms in eukaryotes, and in eukaryotes and prokaryotes, respectively [139,153,154,155,156]. The RNA acts as a template platform for the reverse transcriptase enzyme to express a double-stranded DNA (dsDNA). The expressed dsDNA is integrated into the eukaryotic DNA genomic pool. Retrotransposons utilize a copy-and-paste transferring strategy [139,153,154,155,156]. In contrast, as aforementioned, the DNA platforms (class 2 TEs/DNA Tns)—in both eukaryotes and prokaryotes, including pathogenic microorganisms like bacterial pathogens of *P. aeruginosa*—recruit the mechanism of cut-and-paste [139,153,154,155,156].

The presence of ISs plays a pivotal role in shaping the bacterial accessory genome, such as *P. aeruginosa*. The ISs determine the direction of gene sequestration in genetic exchanges such as HGT [44,148,157]. ISs, as the simplest TEs, are DNA segments with 0.7–2.5 kbp in size compacted with 1–2 *orfs* [(*tnp*) genes] encoding the key enzyme of transposase (Tnp) and a pair of TIRs with a length of 9 to 50 bp (Figure 1) [139,148,158]. Tnps catalyze the vital processes of DNA cleavage, DNA excision, and strand-transfer from donor DNA to the target DNA [148,158]. So, ISs bear conserved short imperfect TIRs adjacent to the left and right sides of the *orfs*. Insertion of the ISs into the target DNA is achieved via the construction of a small flanking DR duplication on insertion [148,158]. Those TEs comprising ISs bear different types of ARGs (including a wide range of β-lactamases/ACGs) or VGs. Depending on the gene’s content, the ISs’ sizes may reach several thousand bps in length [5,28,139,148].

Some ISs contribute to Programmed Ribosomal Frameshifting (PRF) (like ribosome slippage) and Programmed Transcriptional Realignment (PTR) (like RNA polymerase slippage). These mechanisms facilitate the efficient assembly of diverse functional protein domains, enabling a single DNA segment to encode two distinct proteins [44,158,159]. Due to this knowledge, ISs are recognized as the most abundance-independent TEs, which have a pivotal role in the composition of the bacterial cell adaptive genome [44,148]. The insertion of ISs into the bacterial genomes turns them into mosaic-structured systems, which are composed of a core genome interspersed with a variety of accessory genes, like foreign DNA segments, named as mobilome (Figure 1) [160,161].

Ints are effective GEs which have a pivotal role in the assembly and spread of ARGs among bacterial cells, including pathogenic and commensal ones. These ancient GEs have been identified as important resources for bacterial genomic mosaicism and adaptation in both nature and clinical settings [102]. Up to date, the Ints are categorized into five classes, including classes 1–5, in which class 1 Ints are considered as important GEs in clinical settings. According to previous studies, class 1 Ints are key vectors for different types of resistance gene cassettes, which involve resistance features to the majority of antibiotic families [162]. The content of strain-related gene cassettes of Ints is highly variable, so the majority of the cassettes can be identified only in a single Int. The recorded reports depict high dynamicity among cassette arrays, so different levels of variability can be detected within the cassette arrays in association with different environmental conditions [29,102]. Indeed, a gene cassette is a small MGE with a size of 0.5–1 kbp, composed of one (for the most) or two (rarely) genes with no promoter and a recombination site of *attC*. A free gene cassette is a circular non-replicative MGE, which is ready to be inserted to an Int [29,102]. The structure of an Int (chromosomal) is composed of an Int integrase (*intI*) gene [encoding tyrosine recombinase (intI)], an Int recombination site of *attI*, and a cassette array (comprising one or more than 200 sequential gene cassettes) [102]. Following insertion into a mercury-resistance Tn*21*-like transposon, the Tn*402* Tn (carrying an Int) utilizes its *res* site-targeting ability to jump into other MGEs. Through this mechanism, the resulting mosaic element was widely disseminated among various bacterial species [102].

Class 2 Ints, which are related to Tn*7-like* Tns, usually encompass an inactive *intI2* gene, which is associated with the presence of an internal stop codon. Thus, class 2 Ints cover a limited number of certain cassettes [29,163]. In comparison with class 2 Ints, a higher similarity has been detected between class 1 and class 3 Ints. Moreover, it seems that class 3 Ints are related to Tn*402-like* Tns. The majority of the cassettes in class 3 Ints harbor β-lactamase genes [29,164]. Class 4 Ints, also called “sedentary chromosomal Ints” (SCIs), are characterized by giant arrays of cassettes that frequently possess highly similar *attC* sites. It seems that the resistance gene cassettes—though less abundant than other components within SCIs—are the source of those cassettes detected in mobile Ints. Class 4 and class 5 Ints are rare and known as mobile Ints [29,165].

The presence of toxin–antitoxin systems (TASs) supports the stability and maintenance of the conjugative plasmids [113,166]. Indeed, the TASs are ubiquitous small GEs in the genomic pools of bacteria and archaea which are composed of two-gene modules; one produces a stable toxin and another produces the related antidote/antitoxin/mithridate (mithridate is an eponym depicts Mithridates VI Eupator of Pontus: An ancient Iranian Immunologist King who lived in northern Anatolia/Asia Minor/West Asia) to neutralize the expressed toxin [147,167,168,169]. Toxins in TASs are made up of proteins (excluding type VIII TAS, which produces toxins made up of RNAs). In contrast, the produced mithridates/antidotes/antitoxins by TASs are divided into two groups of types I, III, and VIII, which are built of non-coding RNAs, and types II, IV, V, VI and VII, which are proteins [170,171].

TASs are known for their pivotal biological functions in bacterial cells, including plasmid maintenance/persistence (which contributes to persistence of GIs, Tns, ICEs, etc.), virulence, and defense against phage [147,172].

## 6. The Role of ISs, Ints, Tns, Plasmids and TAS in the Transmission of ARGs

In an investigation conducted in Songkhla, Thailand by Chukamnerd et al. [173], the investigators studied thirteen different *P. aeruginosa* sequence types (STs) (fourteen isolates) containing two ST162 isolates, ten different isolates of ST266, ST270, ST313, ST500, ST532, ST647, ST980, ST1097, ST1197, ST1240 and one ST3910 isolate (PA02). Chukamnerd et al. succeeded in detecting and reporting a novel ST3910 isolate from Thailand [173]. Via recruitment of the whole genome sequencing (WGS) technique, and bioinformatics/in silico analysis in a dry lab [174], they [173] identified remarkably high single-nucleotide polymorphisms (SNPs) in the novel ST3910 isolate in comparison with the other isolates. Chukamnerd et al. [173] found 57 ISs in the aforementioned isolates. Moreover, Chukamnerd et al. [173] identified IS*Pa4* and IS*Pa5* as general elements among all isolates. In contrast, the elements of IS*Pa32* (in 12 isolates), IS*222* (in 11 isolates), IS*Pa127* (in 11 isolates), IS*Pa57* (in 11 isolates), and IS*Pa5* (in 10 isolates) were detected in most of them [173]. The ISs of IS*Psy20*, IS*Psy29*, IS*Vapa3*, IS*Pa11*, IS*Pa125*, IS*Pa63*, IS*Pa67*, IS*Pa82*, IS*Pa94*, IS*Pa97*, IS*Pa103*, IS*Aav1*, IS*Ppu27*, IS*Cfr25* and IS*Gpr3* were detected only in one isolate [173]. So, they could find different IS arrays in association with different sequence types. TEs’ target sites are specified in association with the type of TEs [139,175]. DNA Tns are mobilized through a three-step Tnp-driven process involving excising the Tn at its TIRs, cleaving the target DNA to produce sticky ends, and ligating the Tn into the target site. Then, the bacterial host DNA polymerases fill any gaps resulting from this insertion [139,175]. That is why a Tn like Tn*7* attaches to *att*Tn*7* as its specific site within the bacterial chromosome [139,175].

In the study of Che et al. [113], they discovered a huge and complicated network made of IS-related ARGs transfers. Discovering this strong and complicated network underlines the important role of conjugative plasmids and phylogenetically distant pathogens [113]. Hence, the conjugative plasmids and the related ISs are not only involved in a universal evolutionary mechanism for the transmission of ARGs through the HGT feature, but also in the enhancement of genetic exchanges of ARGs in complex communities of microorganisms like biofilms [113]. ISs are at least classified into 27 families [148,158,176]. Among ISs families, IS*26* (63.1%), ISAs*17* (13.55%), and IS*Ecp1* (7.14%) have been recognized as the top three IS families in association with ARGs transfer [148,177].

The results of a scientific project conducted in Warsaw, Poland by Empel et al. [178] showed that the locus of the *bla*_PER-1_ gene in the isolates encompassing the *bla*_PER-1_ gene was situated in Tn*1213* (Tn*4176*). This result confirmed the results reported by previous studies relating to those strains harboring the *bla*_PER-1_ gene from France, Turkey, Belgium, and Italy [178,179,180].

In another study by Papa-ezdra et al. [181], they detected two novel resistance regions in two clinical isolates of *P. aeruginosa* Pa873 (belonging to ST395) and Pa6415 (belonging to ST463) strains taken from ICU patients in Uruguay. ST395 and ST463 are listed as high-risk clones regarding MDR/extensively drug-resistant (XDR) *P. aeruginosa* [182,183]. Papa-ezdra et al. [181] found two novel multi-resistance regions located in a Tn3 family member of IS*Pa40*. Although Tn*3* family members are highly related to ARGs dissemination in both *Pseudomonales* and *Enterobacterales*, recently, the IS*Pa40* has not been reported as one of the most frequent ISs [181,184].

Generally, the IS*26* family members contribute to rearrangement and dissemination of ARGs in chromosomes and plasmids of Gram-positive (like *Clostridium* spp., *Enterococcus* spp., *Listeria* spp., and *Staphylococcus* spp.) and Gram-negative bacteria (like clinical isolates of *P. aeruginosa* and *Enterobacterales*’ bacterial strains) [148,185]. Now, we know three routes and two mechanisms for IS*26*, including copy-in (replicative route by generating and duplication of one more IS copy and the target site, respectively) as the first mechanism and targeted conservative and homologous recombination (as the second mechanism) to cointegrate formation between donor and target DNA molecules [185,186]. The IS*26* is known as an effective participant in association with the highly rated spread of ARGs. Pseudo-compound transposons (PCTns) are significant structures employing a gene or several genes as jumping genes for further dissemination of ARGs to new loci [186]. PCTns are composed of DR-IR-*orf*(*Tnp*)-IR-ARG-IR-*orf*(*Tnp*)-IR-DR in which the ISs are in direct orientation [148,187]. Due to this fact, the PCTn is transferred via a translocatable unit (TU) containing one IS*26*. Hence, IS*26*, as a valuable TE, is able to induce overexpression of adjacent resistance genes such as ARGs [148,186]. As aforementioned, IS*26* family members, rather than IS*26*, such as IS*Sau10*, IS*1216*, IS*1006*, IS*257*/IS*431*, and IS*1008* contribute to ARGs dissemination among both Gram-positive and Gram-negative pathogenic bacteria [186].

Wang et al. [177] performed an effective comparison between the frequency of the ISs- and Int-mediated ARG transfers across the plasmids. They found that the frequency of Int-mediated ARG transfer was remarkably higher than that of ISs on the plasmids [177]. This valuable finding shows that, despite the importance of ISs in the transmission of ARGs via HGT, Ints play a much more important role in the transfer of ARGs on the plasmids [177]. Reported results obtained from previous studies reveal the effective contribution of ISs as well as Ints in bacterial genome rearrangement and transmission of ARGs among plasmids and chromosomes and between chromosomes as well [177]. Due to this knowledge, plasmids recruit ISs and Ints as the most important means to transfer ARGs like ACGs. Thus, superbugs including CRPA, carbapenem-resistant *Acinetobacer baumannii* (CRAB), carbapenem-resistant *Klebsiella pneumoniae* (CRKP), and generally carbapenem-resistant *Enterobacterales* (CRE) recruit plasmid-borne CRGs, e.g., *bla*_KPC_ and *bla*_OXA-48_ to disseminate AMR features among clinical strains [20,177,188,189]. It is known that some of the MDR bacteria are able to bear several plasmids at once. This feature facilitates the exchange of harbored ARGs by plasmids. The simultaneous presence and co-existence of multiple plasmids within a bacterium make them compatible plasmids, while the incompatible plasmids do not obey this rule. The transmission of > 88% of ARGs occurs among compatible plasmids [177]. Wang et al. [177] showed that the frequency of ARGs transmission among compatible plasmids is significantly higher than that of incompatible plasmids. In this regard, 87.9% of ARGs transmissions were detected among compatible plasmids, while only 12.1% of ARGs transmissions were detected among incompatible plasmids [177]. It should be noted that environment-derived plasmids can be acquired by clinical pathogens. Walsh et al. [190] showed that the transmission of environmental-derived plasmids bearing ARGs like ACGs, including *bla*_NDM-1_, into human pathogenic bacteria may cause infectious diseases in the human host body. The source of the *bla*_NDM-1_ gene was drinking water and seepage samples in New Delhi, India. This feature raises great concerns in association with ARGs dissemination among ESKAPE and/or ESKAPEE pathogens such as *P. aeruginosa* [177,190]. As aforementioned, plasmids are recognized as effective genetic platforms that play a pivotal role in the acquisition and dissemination of a wide range of ARGs, such as ACGs (including blaVIM and blaKPC) and quinolone resistance, among different pathogenic bacteria like CRPA and MDR *P. aeruginosa* [113,191,192,193].

In a study performed by Brovedan et al. [194], the authors isolated 13 carbapenem-resistant clinical isolates of *P. putida* from inpatients in Argentina. These isolates encompassed class 1 Int/Tn bearing a cargo of *bla*_VIM-2_ MBL gene cassettes [194]. These cassettes are mostly situated on plasmids or the bacterial chromosome [28,194,195]. In recent years, an emerging resistance to carbapenems together with transmission of ACGs such as *bla*_DIM_, *bla*_IMP_, *bla*_VIM_ and *bla*_NDM_ has been reported among *P. putida* groups [194].

The *bla*_VIM-2_ is the most frequent MBL gene identified in the accessory genome of global clinical isolates of *P. aeruginosa* and in particular among the high-risk clones with MDR phenotypes [28,195]. Brovedan et al. [194] showed the presence of Tn*402*-like class 1 Int harboring *bla*_VIM-2_ gene, which was carried by the conjugative pLD209-related plasmid. Hence, the ACGs, e.g., *bla*_VIM-2_ genes, are able to transpose via conjugative plasmids between the bacterial pathogenic cells, including *P. aeruginosa* and *P. putida* groups [194].

Zhang et al. [196] performed an investigation on the dissemination of the *bla*_IMP-45_ gene among CRPA via a collection of CRPA isolates from patients in China. Indeed, the *bla*_IMP-45_ gene, which is a variant of *bla*_IMP-9_, has been detected as one of the predominant MBLs among clinical isolates of CRPA in China [197,198,199]. The IncP-2 plasmid has a versatile number of sub-lineages, including plasmid pOZ176 (~501 kbp), which carries the *bla*_IMP-9_, *bla*_OXA-10_ and *aacA4* genes as cargos [200,201]. Indeed, IncP-2 plasmids are known as megaplasmids in a single copy which are detectable in different environmental habitats. IncP-2 elements are one of the most common plasmids among clinical isolates of *P. aeruginosa* [201]. Xiong et al. [201] analyzed the IncP-2 carbapenem-resistant plasmid, pOZ176 in their study. Due to this knowledge, they [201] detected two types of class 1 Ints including a Tn*402*-type Int in Tn*6016* and a *sul1*-type Int in Tn*6217* in *P. aeruginosa* 96 (PA96) isolates in China. The Tn*6016* (a Tn*402*-like Int) carried a drug cassette of *aacA4*-*bla*_IMP-9_-*aacA4*. This cassette encodes resistance to aminoglycosides and carbapenems (MBLs) [201]. The Tn*21-like* was located upstream of the cassettes. The second Int, that is, Tn*6217*, carried another drug cassette of *aacA4-catB8a-bla_OXA-10_*. The latter drug cassettes encoded resistance to aminoglycosides, chloramphenicol, and carbenicillin, respectively [201]. The Tn*1403-like* was situated on the upstream of the cassettes. Downstream of the *aacA4-catB8a-bla_OXA-10_* cassettes, an IS*6100* was detected [201]. Now, it is known that the megaplasmid of pOZ176 (~501 kbp) can be involved in both VGs and ARGs, including CRGs transmission in outbreaks caused by the PA96 strain [201].

In a study conducted by Fang et al. [202], they isolated a PA30 strain (an isolate belonging to the ST463 clone) from a male patient in China. The ST463 clone, a high-risk sequence type that spreads quickly, is frequently associated with plasmids that carry the *bla*_KPC-2_ gene [183,202]. According to surveillance conducted on *bla*_KPC_-producing *P. aeruginosa* (KPC-PA) isolates from various hospitals in East China, ST463 has emerged as the predominant CRPA clone [203]. This clone makes up 70.9% of the 151 KPC-PA strains identified in the region [202,203]. Fang et al. [202] detected two plasmids of pPA30_1 and pPA30_2. The IncP-2 megaplasmid [pPA30_1 (~453 kbp)] bears two MBL encoding genes of *bla*_IMP-45_ (IMP-45) and *bla*_AFM-1_ (AFM-1) in an XDR *P. aeruginosa* PA30 isolate. Their analyses [202] showed a high similarity between plasmid pPA30_1 and plasmid pHS17-127. The IncP-2 megaplasmids encompass a similar core genetic backbone and a variable (flexible) AGRs region [202]. The Tn*1403-like* was detected as the MDR determinant in pPA30_1 encoding *bla*_IMP-45_ in *Pseudomonas* spp. Moreover, Tn*1403-like* is highly similar to Tn*6485e* in pHS17-127. The *bla*_IMP-45_ gene located in In*786* (a class 1 Int) carrying five drug resistance cassettes including *aacA4*-*bla*_IMP-45_-*gcu3*-*bla*_OXA-1_-*catB3*. These cassettes are associated with resistance to aminoglycosides, carbapenems, and chloramphenicol [202]. Furthermore, they found two copies of the *bla*_KPC-2_ gene on the second plasmid of pPA30_2 [202]. In addition, Fang et al. [202] found that the accessory regions of pPA30_2 (~49 kbp) were composed of two IS*26* elements arranged as IS*26*-*bla*_KPC-2_-IS*26* genetic platform, originated from Tn*6296*.

In an investigation conducted by Che et al. [113], the investigators clearly showed that ISs play a remarkable role in the dissemination of ARGs via the genetic platforms of conjugative plasmids. In addition, conjugative plasmids are effective platforms for the majority of plasmid-borne ARGs. These ARGs are often located on class 1 Ints [113]. Wang et al. [177] showed that the IS*26* family is the number-one IS family in the dissemination of ARGs. In addition, Che et al. [113] showed that the IS*6* family also has a close association with a variety of ARGs. The ARGs’ accumulation is facilitated by the mobility of conjugative plasmids. Conjugative plasmids are also involved in the dissemination of ARGs in phylogenetically distant bacteria. Indeed, conjugative plasmids are effective genetic platforms for transferring ARGs into an extremely broad spectrum of bacterial recipients. Additionally, these plasmids facilitate genetic exchanges between other plasmids and chromosomes [113,204,205]. According to related studies, a successfully transferred conjugative plasmid is defined by its stable maintenance and/or integration into a chromosome [206].

Weiser et al. [207] accomplished a pan-genomic analysis regarding the industrial *P. aeruginosa* strain RW109. The results obtained from the current study indicated that this strain encompasses a pan-genome of 7,756,224 bp (~7.76 Mbp) in size. The importance of the current investigation is the identification of a megaplasmid with a size of 555 kbp. Further analysis conducted by Weiser et al. [207] revealed that the origin of this 555 kbpmegaplasmid is conserved. This characteristic of the current megaplasmid highlights its role in ecological adaptations, such as thriving in stressful environments, rather than influencing bacterial phenotypic traits.

Megaplasmids not only belong to industrial strains, but also to clinical and pathogenic strains of *P. aeruginosa*. Due to this fact, in a study performed by Cazares et al. [109] in 2020, the investigators isolated 48 clinical strains of *P. aeruginosa* from patients in Bangkok, Thailand. They identified two megaplasmids of pBT2436 and pBT2101 in the genomic pool of clinical strains of *P. aeruginosa*, encoding MDR features [109]. So, these megaplasmids which bear a wide range of cargos including accessory genes, singletons, and other GEs contribute to the ecological lifestyle of the bacterial cells of *P. aeruginosa* to disseminate AMR features among the other strains [109].

In 1983, the *ccdA-ccdB* TAS was identified for the first time, located on an IncF plasmid. This TAS was recognized as a plasmid (e.g., conjugative plasmid) maintenance and persistence system, with a determined function of post-segregational killing (PSK) of plasmid-free bacterial cells [147,206,208,209]. Therefore, the presence of TAS genes guarantees the survival of bacterial host cells. When the plasmid disappears, the produced mithridates/antitoxins/antidotes become neutralized (degraded), and in contrast, the produced toxins kill the plasmid-free bacterial cells. Thus, this phenomenon is named as “plasmid addiction” or “plasmid maintenance/persistence” [147,208]. The plasmid addiction feature enhances vertical inheritance of their replicon [208,210]. By the time more plasmid-borne and chromosomal TASs have been identified in bacteria [172,211]. Hence, TAS can be obtained via MGEs, including phage and plasmids, and they are detectable in bacterial core genomes [172,211]. This phenomenon depicts that TAS can be transferred between pathogenic bacterial cells like CRPA to provide a wide range of advantages, like the acquisition of ARGs like CRGs [147,212]. The TASs classification is based on the mithridates’/antidotes’/antitoxins’ functions and nature. In accordance with the latest classification, TASs are classified into eight types, I to VIII. Due to this classification, except for type I and type VIII TASs, all types of TASs are organized as operon genes [208]. TASs, including type II TAS, contribute to different bacterial characteristics such as environmental adaptation (e.g., AMR phenomenon and antimicrobial tolerance), biofilm formation, Bφ resistance, and virulence [147]. A wide range of studies have been performed in association with type II TAS, and we are aware of its presence in bacterial core genome (chromosomes), and accessory genome [MGEs, e.g., plasmids and PAIs [147]. The results obtained from previous studies show that pUM505, a plasmid detected in *P. aeruginosa*, bears *pumAB*, which is known as plasmid-mediated TAS. This TAS is associated with plasmid stability and virulence enhancement of *P. aeruginosa* [213]. ORFs in PAGI-52 and PAGI-53 (ORF1) encode Type II TAS modules [60].

## 7. Genomic Islands (GIs)

**Figure 2:**
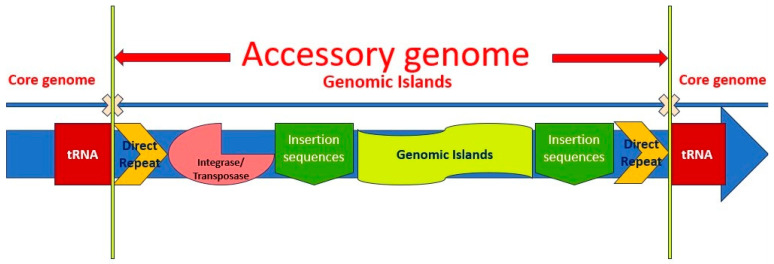
A schematic structure of genomic islands (GIs) as a portion of the accessory genome within the core genome. Accessory genomic pool contains direct repeats (DRs), integrase (Intr)/transposase (Tnp), insertion sequences (ISs), and genomic islands (GIs); while *tRNA* genes are a part of the core genome.

GIs are acquired large clusters of genes that can be transferred via HGT. GIs play a pivotal evolutionary role among bacteria, e.g., *P. aeruginosa*. GIs may be presented in the form of a large bacterial GE with a versatility of contiguous genes through an HGT-mediated insertion [214]. Normally, GIs are integrated into *attB*; a specific site of bacterial chromosomes, which is identifiable by the GI-encoded integrase (Intr) [143,215]. There are some certain characteristics in association with PAGI including atypical GC content in the main chromosomal strains, insertion into the chromosomal region in adjacent to the 3′ end of a *tRNA* gene, flanking by ISs or DRs, armed with a wide range of effective genes ranging from jumping genes like integrase (*intr*) and transposase (*tnp*) genes to VGs, ARGs, MGs or/and other adaptative genes (Figure 2) [48,49,50,51,52,55,59,60,216,217].

The size of GIs normally ranges from 10 to hundreds of kbp (GILs: 10 < bp and GIs: >10 up to 200 kbp) [66,67]. They bear a versatile set of genes with different activities or gene sets, including conjugative genes (e.g., ICEs) and/or viral genes (e.g., a prophage like a temperate phage), which support the transmission of GIs between the bacterial cells [64,143,144]. Some of GIs like GILs (10 < bp) (immobile and those that previously were mobile but not anymore) act as microsatellites; in other words, these GILs lack their own transfer genes and therefore a helper such as a phage is needed to be involved, providing gene products for transmission enhancement [64,66,143,144].

As aforementioned, GIs are normally inserted in adjacent to *tRNA* genes (such as *tRNA^Lys^* and *tRNA^Gly^* genes, etc.) flanked by DRs and based on the phenotype/s (pathogens and non-pathogens) that they encode, are categorized into PAIs, REIs, fitness islands (FIIs), symbiosis islands (SYIs), metabolic islands (MEIs) (Figure 2) [13,29,66,218,219].

Thus, GIs are effective genomic vehicles bearing a wide range of VGs (PAIs) and resistance genes (REIs), which support the adaptation of the bacteria in exposure to different environments and conditions [5,13]. Hence, different strains of *P. aeruginosa*, including CRPA, are able to acquire a wide range of foreign genes through a single event of HGT [13]. A single genomic locus can independently acquire multiple GEs, resulting in what is known as a “mosaic” GI, regardless of the diverse genomic environments from which the elements originated [214]. Therefore, GIs are disseminated among different types of bacterial strains via intercellular or intracellular mechanisms of HGT, including transformation, transduction, conjugation, and vesiduction [28,220,221,222]. Moreover, it should be considered that GIs carry those genes contributing to mobility, such as *Intrs* and *Tnps*, etc. (Figure 2) [13,28].

The GIs are capable of integrating into or excising from the bacterial chromosomes [28]. As aforementioned, those GIs that bear ARGs are known as REIs. MDR, XDR, and pandrug-resistant (PDR) bacterial strains, including *P. aeruginosa*, are considered a serious global concern. In this regard, a wide range of ARGs are disseminated via REIs among vancomycin-resistant *Enterococcus* (VRE), methicillin-resistant *S. aureus* (MRSA), extended-spectrum β-lactamase (ESBL)-producing *Enterobacterales*, CRAB, MDR/XDR/PDR *A. baumannii*, CRE, CRKP, MDR/XDR/PDR *P. aeruginosa*, and CRPA [1,2,5,6,7,28,182,223]. MDR, XDR, and PDR terms are defined as non-susceptible to ≥one agent in ≥three antimicrobial groups; susceptible with limitation of ≤two antimicrobial groups; and non-susceptible to all agents in exposure to all antimicrobial groups, respectively [224].

GIs/ICEs play a remarkable role in the resistance in high-risk clones of *P. aeruginosa*. So, they bear cassette-borne genes within class 1 Int/Tn for the most part, and sometimes inserted within the Tn*21* subfamily Tns [29,225]. ICEs in *P. aeruginosa* are classified into two main families, including *clc*-like ICEs and pKLC102-related ICEs (a plasmid-origin GI). *clc* is the initialism for chlorocatechol, which is related to acquired genes for chlorocatechol degradation [13,28,55,226].

As the pKLC102 is capable of acting like a free multicopy plasmid or a GI in some isolates of *P. aeruginosa* strains, it is recognized as a plasmid. A part of pKLC102 may be identical to a smaller plasmid bearing a class 1 Int [29,51].

The population structure of *P. aeruginosa* is both panmictic and epidemic. Due to this fact, the observed level of recombination is high, and loci demonstrate random association [227,228]. Bacterial populations under panmixia, or random mating, show a state of linkage equilibrium and maintain high genetic diversity at neutral sites in their genomes [228]. Virulence, antimicrobial resistance, site-specific pathogenicity, and specific infection outcomes are commonly linked characteristics to high-risk clones, which are identifiable by their unique sequence types [229]. It seems that “high-risk clones” of *P. aeruginosa* are not simply random isolates, but rather genetically distinct lineages. Their ability to enhance the acquisition of resistance genes is associated with the presence of different MGEs. In an investigation performed by Correa et al. [230], they showed that the XDR CRPA, including the high-risk clone ST111, harbors *bla*_VIM-2_ within a class 1 int and the high-risk clone ST235 harbors *bla*_KPC-2_ within Tn*4401* Tn. They also found a novel variant of ST111, *P. aeruginosa* ST1492 [230]. In a study conducted by Nageeb et al. [228], they used Multi-Locus Sequence Typing (MLST) to accomplish an analysis on a population of 528 *P. aeruginosa* sequences. Nageeb et al. [228] could detect 249 STs, of which 229 STs were known, while 20 20STs were novel. In this regard, the top 13 international ST clones from 1 to 13 were ST235 [serotype O11 (98%)], ST111 [serotype O12 (83.3%) and serotype O4 (16.7%)], ST244 [serotype O12 (53.9%) and serotype O2 [58.5%)], ST308 [serotype O11 (100%)], ST395 [serotype O6 (100%)], ST253 [serotype O10 (92.3%)], ST348 [serotype O2 (88.9%)], ST274 [serotype O3 (100%)], ST179 [serotype O6 (90%)], ST233 [serotype O6 (100%)], ST17 [serotype O1 (100%)], ST27 [serotype O1 (100%)] and ST175 [serotype O4 (100%)], respectively [228]. Nageeb et al. [228] identified a new set of molecular markers linked to globally prevalent high-risk *P. aeruginosa* clones. Their research [228] also indicated that the prevalence of these high-risk clones correlates with the presence of MGEs and chromosomal variations, suggesting their importance as risk-associated markers.

Del Barrio-Tofiño et al. [182] published a review to prepare a 2020 update on nosocomial MDR/XDR high-risk *P. aeruginosa* clones. The results presented the top ten *P. aeruginosa* high-risk clones comprising ST235, ST111, ST233, ST244, ST357, ST308, ST175, ST277, ST654, and ST298, which are MBL producers. The MDR *P. aeruginosa* clones of ST111, ST175 and ST235 bear REIs. As the previous reports show, the *P. aeruginosa* high-risk clones of ST111 and ST235 are considered the most CRPA strains associated with production of horizontally-acquired β-lactamases, including classes A, B, and D carbapenemase enzymes [28,182,195]. Moreover, it is noteworthy to have specific attention on ST235 as the highest-risk clone, which contributes to the dissemination of > 60 different β-lactamases (comprising a wide range of carbapenemases from classes A and B on a global scale) [182]. According to the recorded reports, the MDR *P. aeruginosa* clones of ST111, ST175, and ST235 employ a wide range of MGEs such as GIs/RIs, Tns, Ints, and plasmids to transfer ARGs, e.g., carbapenemase genes from one bacterial cell to another [28,182,225]. All in all, the ACGs are disseminated via Ints, GIs/RIs, Tns, and plasmids for the most part [13].

In a recent study published by Flores-Vega et al. [231], the authors present some global MDR/XDR *P. aeruginosa* high-risk clones, including ST111, ST175, ST233, ST235, ST244, ST274, ST395, and ST463 harboring β-lactamase genes contributing to chronic colonization and increasing infections as well as morbidity and mortality among patients. In this regard, MLST as an effective identifiable strategy and global standard methodology has been used to detect high-risk clones and to achieve a rigorous molecular epidemiology investigation in association with *P. aeruginosa* [231]. The PubMLST (https://pubmlst.org/organisms/pseudomonas-aeruginosa, accessed on 22 March 2025), a public database for molecular typing and microbial genome diversity, is available [232]. Because of the importance of CRPA strains and WHO’s definition as a high priority [11], we discuss them according to the reported results by Flores-Vega et al. [231] in brief, as below:

**ST111:** The ST111 clone of *P. aeruginosa* AG1 (PaeAG1), an O12 serotype strain, exhibits a global distribution, having been detected across all continents, such as North, Central, and South America; the Middle East (West Asia), the Persian Gulf region, India, and Southeast Asia; Northern, Southern, Eastern, and Western Europe; Africa; and Oceania (e.g., Australia). The ST111 XDR phenotype has been recognized as a high-risk clone. The ST111 strain harbors different variants of β-lactamase genes belonging to classes A (involving *bla*_CTX-M-like_, *bla*_GES_, *bla*_KPC_, *bla*_PSE_, *bla*_TEM-like_, and *bla*_VEB_), B (including MBLs and carbapenemase enzymes, e.g., *bla*_GIM_, *bla*_IMP_, and *bla*_VIM_), and D (*bla*_OXA_). The clinical ST111 isolates often harbor *bla*_VIM-2_ and *bla*_IMP-18_ within a class 1 Int situated in 57 PaeAG1 island [145,231,233,234].

**ST175:** The ST175 is a high-risk clone which belongs to serotype O4. The main portion of ST175 clones encompasses the MDR phenotype and is armed with *bla*_VIM-2_. As previous studies show, the MBLs and carbapenemase genes, e.g., *bla*_VIM-2_ gene and other resistance genes such as *aac6’-1b* (encoding aminoglycoside modified enzyme) make drug cassettes within a class 1 Int, which is usually situated on Tns, plasmids, or chromosomes. The overexpression of AmpC and efflux pump MexXY, high resistance to fluoroquinolones, and overexpression are often detectable among ST175 XDR phenotype. The distribution of ST175 clones encompasses North America, Europe (central and southern regions), and Asia, including the Persian Gulf region and Southeast Asia. Compared with ST111, the ST175 strain harbors narrower spectrum variants of β-lactamase genes belonging to classes A (involving *bla*_AER_, *bla*_CARB_, *bla*_GES_, and *bla*_TEM-like_), B (including MBLs and carbapenemase enzymes, e.g., *bla*_IMP_, and *bla*_VIM_), and D (*bla*_OXA_) [145,194,200,201,231,234,235,236].

**ST233:** ST233 is a high-risk clone which belongs to serotype O6. The clone K34-7 is a carbapenem-resistant isolate that belongs to ST233. The ST233 XDR phenotype expresses MBLs and carbapenemase enzymes via harboring variants of β-lactamase genes belonging to classes A (*bla*_GES_), B (including MBLs and carbapenemase enzymes, e.g., *bla*_IMP_, *bla*_NDM,_ and *bla*_VIM_), and D (*bla*_OXA_). The ST233 clone has spread globally, reaching every continent: North, Central, and South America; the Middle East (West Asia), the Persian Gulf region, India, and Southeast Asia; all regions of Europe (North, South, East, and West); Africa; and Oceania (Australia) [145,231,234,237,238,239].

**ST235:** The ST235 high-risk clone has been identified as the most frequent isolate across the world. ST235 clone is armed with > 60 variants of β-lactamase genes from different classes of A (involving *bla*_BEL_, *bla*_CTX-M_, *bla*_GES_, *bla*_KPC_, *bla*_PER_, *bla*_PSE_, *bla*_SHV_, *bla*_SMP_, *bla*_TEM_ and *bla*_VEB_) B (including MBLs and carbapenemase enzymes, e.g., *bla*_FIM_, *bla*_IMP_, *bla*_NDM_ and *bla*_VIM_), and D (like *bla*_LCR_, *bla*_NPS_, *bla*_OXA_). According to previous studies, the ST235 clone, together with other high-risk clones involving ST298, ST357, and ST308, belongs to serotype O11. The ST235 clone has spread globally, with documented dissemination across all continents: America (North, Central, and South), Asia (including the Middle East (West Asia), the Persian Gulf region, India, and Southeast Asia), Europe (North, South, East, and West), Africa, and Oceania (Australia) [145,173,231,234,240].

**ST244:** Although the ST244 is not capable of expressing the *bla*_OXA-74_ gene, there are no considerable differences between ST244 and ST235 clones. In addition, the ST244 clone is associated with two different serotypes of O2 and O12. The ST244 MDR phenotype, as the high-risk clone, possesses different variants of β-lactamase genes from different classes of A (containing *bla*_AER_, *bla*_CTX-M_, *bla*_KPC_, *bla*_SHV-like_, *bla*_TEM,_ and *bla*_VEB_) B (including MBLs and carbapenemase enzymes, e.g., *bla*_IMP_, *bla*_NDM,_ and *bla*_VIM_), and D (*bla*_OXA_). ST244 clones are found throughout North and South America, Europe (northern, southern, eastern, and western regions), and Asia, notably in the Middle East (West Asia), the Persian Gulf area, India, and Southeast Asia [145,178,231].

**ST274:** ST274, classified as an international epidemic high-risk clone, is endemic to Spain and belongs to serotype O3. Globally, ST274 is a major hospital-associated clone linked to CF in patients. MDR and XDR ST274 phenotypes are recognized as high-risk clones. The variants of β-lactamase genes in ST247 clone are limited and include some variants from different classes of A (e.g., *bla*_AER_ and *bla*_GES_), B (including only one MBL and carbapenemase enzyme, e.g., *bla*_IMP_), C (such as *bla*_PDC_ and *bla*_PAO_), and D (*bla*_OXA_). Notably, this clone incorporates variants of the C class β-lactamase genes. ST274 clones exhibit widespread distribution across North and South America, Europe (central and southern regions), Asia (the Middle East, the Persian Gulf region, and Southeast Asia), Africa, and Oceania (Australia) [145,231,241].

**ST395:** The international, MDR ST395 clone, a high-risk lineage known worldwide, is considered endemic in France. This clone is characterized by a 131 kb chromosomal deletion impacting genes involved in bacterial adhesion, biofilm formation, and type IV pili biosynthesis. This deletion may contribute to reduced bacterial pathogenesis. ST395 encompasses different variants of β-lactamase genes from different classes of B (including MBLs and carbapenemase enzymes, e.g., *bla*_VIM_) and D (*bla*_OXA_). These genes may be situated in class 1 Int in the cassette of *intI1*-*bla*_OXA-10_-*aadB*-*bla*_VIM-2_-*aadB*-*bla*_OXA-10_. In addition, the resistance genes of *aadA6*, *aph(3′)-IIb*, and *aph(3′)*-via and *catB7*, have been detected in the genomic pool of ST395 clones. ST395 bears type I-E CRISPR/Cas system, too. Detection of ST395 dissemination has been limited to a specific geographical region, involving areas in North America, Europe (northern, southern, eastern, and western regions), and Africa [145,231,242,243,244].

**ST463:** ST463, an XDR clone belonging to serotype O4, harbors various β-lactamase gene variants, including class A (*bla*_KPC_), class B (containing MBLs like *bla*_IMP_ and *bla*_AFM_), and class D (*bla*_OXA_). Acquisition of the *bla*_KPC-2_ gene further enhances the resistance of this ST463 clone to carbapenems and other β-lactam antibiotics. Hu et al. [245] isolated a CRPA strain, PA1011 (ST463), from a patient in China. This strain harbored a ~63 kbp-plasmid, pPA1011, carrying the *bla*_KPC-2_ carbapenemase gene. While plasmids pPA1011 and p14057 (~53 kbp) share some similarities, pPA1011 is characterized by unique regions encoding phage-related and hypothetical proteins, as well as a diverse array of MGEs. These MGEs include IS*Kpn27*, IS*Kpn6*, a Tn*3-like* Tn, and two copies of IS*26* within the cassette ΔIS*6*-Tn*3*-IS*Kpn8*-*bla*_KPC-2_-IS*Kpn6*-IS*26*. It is also important to note that ST463 is recognized as a significant endemic clone in China and Korea. [145,183,231,245].

Although 90% of the *P. aeruginosa* chromosome is conserved, the remaining 10% belongs to the accessory genome, in which GIs are a portion of its composition [50,246,247,248].

The results obtained from several important studies have revealed 53 GIs among *P. aeruginosa* strains, which are presented in Table 1.

According to Table 1, we describe the PAGIs’ structures and functional characteristics briefly as before.

### 7.1. PAGI-1’s Structure and Functional Characteristics

Liang et al. [48] identified and characterized PAGI-1 in *P. aeruginosa*. Consequently, they detected PAGI-1 in 85% of *P. aeruginosa* isolates obtained from patients with UTIs and sepsis. Furthermore, unlike many PAIs, the situation of PAGI-1 was not adjacent to any *tRNA* genes [48]. The PAGI-1 is integrated into the *P. aeruginosa* chromosome by replacing a ~7 kbp region normally found in strains lacking PAGI-1, such as PAO1. PAGI-1 also contained two insertion sequences, potentially facilitating its transfer between bacteria [48]. The PAGI-1 integrates at different locations within the genome, but a ~7-bp region within it is preferentially targeted by other GIs. Additionally, two transcriptional regulators are expressed by the PAGI-1 [48,249]. The expression of these regulators may affect the activity of genes both within PAGI-1 and on the chromosome itself [48,249]. The PAGI-1 appears to be a mosaic structure incorporating modules from at least two distinct sources. This suggests that it was assembled within a previous bacterial host, potentially one unrelated to *P. aeruginosa*. A recombination-driven replacement process ultimately led to the integration and stabilization of PAGI-1 within the chromosome of numerous *P. aeruginosa* strains [48].

### 7.2. PAGI-2’s and PAGI-3’s Structures and Functional Characteristics

To explore the genomic diversity of *P. aeruginosa* clone C, Larbig et al. [49] sequenced a highly variable ~110 kb region in two strains, C and SG17M. This region, situated adjacent to the *lipH* gene, is characterized by significant mosaicism within the bacterial chromosome [49]. Sequencing revealed this region consists of two key components in both strains: a strain-specific gene island {containing 111 ORFs in strain C [PAGI-2(C)] and 106 ORFs in SG17M [PAGI-3(SG)]} and a 7 kb core fragment of *P. aeruginosa* clone C (containing nine ORFs) [49]. A key difference between strains SG17M and C lies in the integration site of their respective MGEs within the *tRNA^Gly^* gene cluster. Strain SG17M shows the integration of PAGI-3(SG) into the first gene, while strain C exhibits integration of PAGI-2(C) into the second [49]. All in all, both PAGI-2(C) and PAGI-3(SG) display a two-part structure. One part is comprised of strain-specific ORFs encoding metabolic enzymes and transport proteins. The other part contains conserved hypothetical genes, and a significant number of genes exhibiting substantial sequence similarity to one another are homologs [49].

### 7.3. PAGI-4’s Structure and Functional Characteristics

Klockgether et al.’s project [51] included *P. aeruginosa* strains from various sources. These included clones C, C17, K, K1, and K2 (clones K and C represented) isolated from cystic fibrosis (CF) patients, as well as strain SG17 M (clone C), which originated from an aquatic environment. Plasmids pKLK106 and pKLC102 have a significant role: they highlight a rare dynamic relationship in which mobile DNA coexists as both a free plasmid and a genome island within a single bacterial cell, potentially impacting gene transfer and bacterial evolution. Klockgether et al. [51] selected the clone C plasmid pKLC102 to sequence, because it is a major clone of the present *P. aeruginosa* population in environmental and disease habitats. A series of phylogenetic analyses and annotations was performed on a large 103,532-bp pKLC102 sequence by Klockgether et al. [51]. The investigators [51] indicated that pKLC102 is a hybrid element, exhibiting characteristics of both plasmids and phages. Klockgether et al. [51] showed that, pKLC102 was disrupted by the irreversible insertion of TNCP23, a large (~23 kbp) class 1 Tn. TNCP23 is a complex GE containing plasmid, Int, and IS*6100*. Transposition of an IS*6100* copy within TNCP23 led to chromosomal inversions and a breakdown of the original plasmid-like arrangement. Klockgether et al. [51] selected cosmid pKSCC260 to be sequenced. The reason was the detection of PAO1-like DNA from the *oprL-phnAB* region at both insert ends. PAGI-4(C) is situated at *tRNA^Lys^* close to *oprL-phnAB*. Therefore, it seems that the PAGI-4(C) gene island is surrounded by pKSCC260. The sequence annotation of pKSCC260, done by Klockgether et al. [51] depicted the integration of PAGI-4(C) *tRNA^Lys^* site in strain C.

### 7.4. PAGI-5’s Structure and Functional Characteristics

Battle et al. [52], identified PAGI-5 in *P. aeruginosa* (PSE), which is predicted to encompass 121 ORFs. The PAGI-5 is integrated adjacent to a *tRNA^Lys^* within the core chromosome. The PAGIs together with other GIs, e.g., PAP-I, PAP-2, etc., are members of the pKLC102-related GIs family. These family members are common in β- and γ-proteobacteria [52,219]. The pKLC102-related GIs family members are plasmid–phage hybrids which have bi-sectional structure including a semi-conserved core set of genes contributing to vital activity like replication and variable “cargo” gene cassettes [51,219,250]. PAGI-5 exhibits the highest similarity to PAPI-1, with 79 out of its 121 predicted ORFs showing resemblance to PAPI-1 ORFs [52].

### 7.5. PAGI-6’s-PAGI-11’s Structures and Functional Characteristics

Battle et al. [50], in another project, detected PAGI-6, PAGI-7, PAGI-8, PAGI-9, PAGI-10 and PAGI-11 in *P. aeruginosa*. PAGI-6 exhibits strong similarity to φCTX, an R-pyocin-related, cytotoxin-converting phage from *P. aeruginosa* PA158. φCTX belongs to the *P. aeruginosa* R-pyocin-related phage family [50]. It is noteworthy that the integration of PAGI-6 into the *tRNA^Thr^* gene occurs in PSE9, while in *P. aeruginosa* strain PA5160.1, the integration of φCTX phage genome into *tRNA^Ser^* gene occurs in *P. aeruginosa* strain PA158 [50,251]. Moreover, Battle et al. [50] found that although the genome of φCTX is smaller than PAGI-6, a large portion of both sequences is relatively well conserved. PAGI-7 is composed of 20 ORFs. Several mobility-associated ORFs, predicted transcriptional regulators, and a predicted *ptxABCDE* operon form PAGI-7 [50]. PAGI-8 is made up of 12 ORFs in which phage Intr and IS*407* are identifiable [50]. PAGI-9 and PAGI-10 are composed of only one ORF, which is very large; however, PAGI-9 is larger than PAGI-10. The PAGI-9’s ORF resembles the rearrangement hs (Rhs) family of GEs. This condition has been detected in PAGI-10 too. Hence, the PAGI-10’s ORF resembles an Rhs core ORF [50]. PAGI-11 has been detected as the smallest among the other PAGIs. Neither ORF nor repeated sequence has been identified in PAGI-11 [50].

### 7.6. PAGI-12’s Structure and Functional Characteristics

Singh et al. [54] identified the PAGI-12 in their study. According to their sequence analysis and sequence homology-based annotation in association with PAGI-12, it is composed of 137 predicted ORFs. Singh et al. [54] showed a significant homology between PAGI-12 and genes from several organisms, including *Xanthomonas* spp., *Rhodopseudomonas* spp., and *Bordetella* spp., etc. Further investigation via BLAST (http://blast.ncbi.nlm.nih.gov/Blast.cgi) homology technology performed by Singh et al. [54] revealed that the main group of genes located in PAGI-12 is associated with different categories, such as DNA recombination, repair, and modification; transcriptional regulation; pathogenesis, e.g., T4SS components; antibiotic and heavy metal resistance; hypothetical protein; two-component response regulator systems, e.g., GacS-like system; metabolism, etc.

### 7.7. PAGI-13’s and PAGI-14’s Structures and Functional Characteristics

Silveira et al. [55], successfully detected PAGI-13 and PAGI-14 in their investigation. They found that [55] PAGI-13 is predicted to have 235 coding sequences (CDSs). Its genetic architecture starts with an *intr* gene, followed by genes grouped into five key categories comprising genes linked to phages/prophages/TEs/plasmids; genes participating in regulation and cell signaling; genes associated with virulence/disease/defense, such as IS*CR14-rmtD* and In*163* genes; genes related to stress response; genes encoding hypothetical proteins [55]. The data suggest that a phage or a plasmid with the ability to integrate is responsible for the formation of PAGI-13 [13,49,55]. Moreover, the presence of the *tRNA^Gly^* gene within PAGI-13 appears to be a hs for MGEs. This feature enhances the bacterial strain’s versatility and simultaneously provides an ecological advantage to this clone in a variety of environments [55]. Silveira et al. [55] showed that PAGI-14 begins at the *tRNA^Pro^* gene, and it does not belong to either the *clc*-like ICE or the KLC102-related ICEs. PAGI-14 is composed of 41 predicted CDS, with a minor portion of these genes encoding proteins like Intr. These proteins contribute to the type III restriction system, regulation, etc.; however, the majority of the 41 predicted CDS in PAGI-14 encode hypothetical proteins [55]. Silveira et al. [55] identified an inserted element of In*163*, at the 5′ end of PAGI-13 in all strains of ST277. They suggested that class 1 In*163* was firstly inserted into the chromosome of the ST277 strains’ common ancestor, and then the integration of the *rmtD* gene (aminoglycoside resistance gene) occurred [55]. Furthermore, the ICEPaeSP was identified only in those ST277 strains harboring *bla*_SPM-1_ gene. Hence, they [55] reported that inserted elements of *rmtD* and In*163* are situated in PAGI-13 while genes in PAGI-14 are responsible to encode those proteins contributing in type III restriction system and phages.

### 7.8. PAGI-15’s and PAGI-16’s Structures and Functional Characteristics

In a study conducted by Hong et al. [56], 431 *P. aeruginosa* clinical isolates were enrolled. The authors discovered that all of the CRPA strains belonged to ST235, and two of the CRPA strains belonged to ST244. Hong et al. were able to detect the gene cassettes of class 1 Ints carrying cargo genes including *bla*_IMP-6_, *bla*_IMP-10_, *bla*_VIM-2_, and *bla*_GES-24_. The gene cassette of In*IMP-6D* was detected as the carrier of the *bla*_IMP-6_ gene within the array of *intI1-bla*_IMP-6_*-qac-aacA4-catB3-aacA4-bla*_OXA-1_*-aadA1*. In an isolate, *bla*_IMP-10_ within the new class 1 Int of In*IMP-10*, was detected. *IMP-10* carries the *bla*_IMP-10_ gene within the *intI1-bla*_IMP-10_-*qac-aacA4-catB3-aacA4-bla*_OXA-1_*-aadA1* array. The *bla*_VIM-2_ gene cassette is carried by different class 1 Int. Hong et al. [56] successfully presented three different gene cassettes and the related arrays, including In*559* (*intI1-aacA7-bla*_VIM-2_*-dhfa-aadA5*), In*VIM-2*_JH5_ (*intI1-aadB-bla*_VIM-2_*-aacA4-orf-ereA*), and In*VIM-2*_JH6_ (*intI1-aacA4-bla*_VIM-2_*-aacA4-fosC-bla*_OXA-2_*-qac*). Moreover, the In*GES-24* identified as a novel class 1 Int as a gene cassette bearing the array of *intI1-aacA4-aadB-bla*_GES-24_*-aacA4-bla*_OXA-2_. Hong et al. detected the class 1 Int of In*IMP-6D* within the PAGI-16. In addition, they [56] identified that PAGI-16 is composed of 99 ORFs, in which 9 of them originate from the *clc*-element. A Bφ-P4-like Intr mediates the insertion of the *clc*-element at the *attB* site, which lies between two tandem *tRNA^Gly^* genes located at the 3′ end [13,56]. Ten genes of 99 ORFs encode Tnp and Intr enzymes and ISs. Eight of the 99 ORFs encode antimicrobial resistance proteins involving *aacA4* (two copies), *aadA1*, *catB3*, *cmx*, *sul1*, and *bla*_OXA-1_ and *bla*_IMP-6_. The rest of the genes in PAGI-16 encode hypothetical proteins [56]. Additionally, Hong et al. [56], detected class 1 Int of In*GES-24* within the PAGI-15 region. Devoid of inserted ICEs, the PAGI-15 and PAGI-16 sequences have a similarity of 99.99%. Hong et al. [56] were able to identify 11 drug resistance genes, including *aacA4* (two copies), *aadB*, *strA*, *strB*, *tet(G)*, *cmx*, *sul1* (two copies), *bla*_OXA-2_, and *bla*_GES-24_ in PAGI-16, while these genes were not detected in PAGI-15. In place of *qacE*Δ*1* at 3′ CS, the In*559* is replaced by *tniC* of Tn*5090* at the 3′ end. This evidence suggests a different evolutionary pathway by the recruitment of Tn*5090* [56]. These astonishing results support the dissemination of different ACGs in *P. aeruginosa* [56].

### 7.9. PAGI-17’s Structure and Functional Characteristics

In an investigation conducted by Abril et al. [57] in Colombia, 27 out of 58 *P. aeruginosa* isolates were detected as CRPA strains. Moreover, 6/27 CRPA strains were armed with the *bla*_VIM_ gene and 4/27 CRPA strains were armed with the *bla*_KPC-2_ gene. Those CRPA strains bearing the *bla*_KPC-2_ gene were identified as belonging to a ST235 clone. Six strains of those CRPA harboring the *bla*_VIM_ gene belonged to different sequence types, containing two isolates of ST111, one isolate of ST244, two isolates of ST1978, and one isolate of ST235. According to genome sequencing analysis of the ST235 *bla*_KPC-2_-positive (24Pae112), Abril et al. [57] identified several different resistance gene cassettes of *ant(2″)-Ia, ant(3″)-Ia*, and *aph(3′)-IIb*, *bla*_OXA-2_ and *bla*_KPC-2_, *sul1*, *catB7* and *fosA*. Furthermore, they identified a mass of ICEs within the antimicrobial resistome of the 24Pae112 chromosome, such as eight prophages [H70 (two copies), PM105 (two copies), D3 (one copy), F10 (one copy/bearing Tns with resistance genes, e.g., Tn*402-like*), φCTX (one copy), Pf1 (one copy)], seven PAGIs [PAGI-1 to PAGI-6 (PAGI-5 bearing ISs, Tns with resistance genes, e.g., IS*Pa40-like*, Tn*402-like-2*), GI1 (bearing Tns with resistance genes, e.g., Tn*6162-like-aadB-gcuD*) and PAGI-17 (bearing Tns with resistance genes, e.g., two copies of Tn*4401b*)] and several Tns [57]. All in all, they detected eight different resistance genes and five Tns, including one Tn*6162-like*-ant(2″)-*Ia*, two Tn*402-like-ant(3″)-Ia-bla*_OXA-2_ and two Tn*4401b-bla*_KPC-2_ [57].

### 7.10. PAGI-18’s Structure and Functional Characteristics

Urbanowicz et al. [58], investigated on the isolate of *P. aeruginosa* 1334/14, which was categorized as ST234. Its antimicrobial resistome was composed of *bla*_NDM-1_, *bla*_DIM-1_, ESBL gene *bla*_PME-1,_ and several aminoglycoside resistance genes, e.g., *rmtD3*. They found that [58] the *bla*_NDM-1_ gene is located in PAGI-18. It seems that the PAGI-18 appeared via a number of rearrangements that occurred by MGEs, such as IS*6100* and In*1592* [58].

### 7.11. A Brief Description Regarding PAGI-15’s-PAGI-40’s Structures and Functional Characteristics

In an investigation conducted by Nascimento et al. [59], the investigators presented effective data and information in association with several PAGIs, including PAGI-15 to PAGI-40. In this regard, some important antimicrobial resistance genes comprising *bla*_SPM−1_, *bla*_OXA−56_, *rmtD*, *cmx*, and *sul1* are located in PAGIs of PAGI-15 and -25. A similarity of 63% has been detected between PAGI-34 and PAPI-1. However, PAGI-34 bears a CRISPR-Cas system which is composed of a collection of genes involving *cas3, cas5, cas8c, cas7, cas4, cas1*, and *cas2* genes (type I-C). As ICEs harbor type I-C CRISPR-Cas systems, PAGI-34 exhibits a high coverage and sequence similarity of 99% and 99%, respectively, to MGEs, e.g., pKLC102-like ICEs [59,252]. HGT is known as a serious threat to bacterial genomes through the insertion of disruptive MGEs like phages and plasmids. To combat this, bacteria employ CRISPR-Cas systems as their adaptive immune systems. Due to this knowledge, CRISPR-Cas systems as functional bacterial adaptive immune systems prevent the integration of MGEs, protecting against disruptive insertions, gene inactivation, and unwanted genetic rearrangements [59,252]. The ARGs, including *bla*_OXA−56_, *aadA7-aac(6′)-Ib-cr*, *sul1*, and *cmx*, are detected in three different MGEs carried by a Tn*As3*. This Tn together with its cargos (ARGs) have been detected as an inserted genetic segment within the PAGI-25 [59].

### 7.12. PAGI-41’s Structure and Functional Characteristics

In another study performed by Espinosa-Camacho et al. [60]. The authors detected PAGI-41, which was composed of 214 ORFs. They found that proteins with unknown functions are expressed by the ORF31 to ORF214. They detected several acquired genes within the PAGI-41, which were integrated via different MGEs, such as pGES5 and pSDENCHOLpb plasmids, a class 1 Int carrying ARGs and HMRGs, Tn*Pa38* carrying genes which contribute to vital activities, some genes involved in defense against viral infections, and Tn3 family *tnp* genes [60].

### 7.13. PAGI-42’s Structure and Functional Characteristics

Espinosa-Camacho et al. [60] identified PAGI-42 and described its structure and characteristics. Thus, PAGI-42 is composed of 200 ORFs in which ARGs, HMRGs, and metal transporter encoding genes are detectable. These genes are integrated into PAGI-42 via Tn*4652*. PAGI-42 is armed with a wide range of Tnps, e.g., the IS3 family (IS*Pa32*, IS*Pa38*) and the IS5 family (IS*Pa95*). At the 3′ end of PAGI-42, a Tn*Pa42* has been detected, which contributes as a copper resistance feature. This Tn has 100% similarity with the Tn located within PAGI-41 [60]. Espinosa-Camacho et al. [60] detected PAGI-43, as the largest PAGI in *P. aeruginosa* up to now. PAGI-43 is composed of 356 ORFs. Of the 356 ORFs, 151 (~43%) are shared between PAGI-42 and PAGI-43 and encode hypothetical proteins [60]. In PAGI-43, the DNA fragment spanning ORF132 to ORF147 is an inverted segment from PAGI-42 and encodes structural components of certain Bφs.

### 7.14. PAGI-43’s and PAGI-44’s Structures and Functional Characteristics

Some regions in PAGI-43, including ORFs 40–131 and 148–259, are known as hybrid sections which are involved in encoding hypothetical proteins, phages’ tail and capsid proteins, etc. [60]. Espinosa-Camacho et al. [60] could also identify the PAGI-44 element. This PAGI is composed of 140 ORFs, of which 44% encode hypothetical proteins. The first 30 ORFs belonging to PAGI-1 have been detected in PAGI-44 too. An inserted Tn of Tn*Pa42*, which harbors aminoglycosides resistance genes, has been detected between ORF16 and ORF17 in PAGI-44. The region spanning ORF55 to ORF140 contains triplicated gene clusters. It seems that ≥ three independent recombination events have occurred in this region [60]. These recombination processes may be the outcome of the presence of a versatile set of Tnps (belonging to Tn*3*, IS*3*, and IS*66* families), carried by phages. ORFs encoding proteins such as TnpS and TnpT, which are involved in cointegration, are found in this region. This suggests a mechanism for increased genetic variation mediated by phages and Tns, which can introduce or remove genetic sequences.

### 7.15. PAGI-45’s and PAGI-46’s Structures and Functional Characteristics

In follow-up research, Espinosa-Camacho et al. [60] found PAGI-45, which involves 63 ORFs. PAGI-45 is made up of PAGI-1’s first 30 ORFs, one of the ISNY family members (IS*Bcen27*), and those ORFs which express hypothetical proteins. PAGI-46 is built of 103 ORFs. The last 60 ORFs in PAGI-46 has a hybrid composition including ORF41 to ORF101 (PAGI-2 block), ORF44 to ORF120 (PAGI-3 block) and ORF46 to ORF107 (Liverpool epidemic strain GI-3 (LESGI-3) block) [60]. LESGI-3 together with *clc* (a GI made up of 105-kbp, which was detected in *P. knackmussii* strain B13, for the first time) itself, PAGI-2, PAGI-3 are known as the *clc*-like ICEs [13]. Sequencing analysis reveals that the initial 40 ORFs of PAGI-46 strongly resemble chromosomal regions of *Burkholderia lata* A05 (75% similarity) and *Bordetella bronchiseptica* F709 (71% similarity), two opportunistic pathogens that are significant sources of infection in immunocompromised patients in the ICU [60,253,254,255].

### 7.16. PAGI-47’s Structure and Functional Characteristics

PAGI-47, a hybrid genetic island composed of 278 ORFs, is divided into two functionally distinct regions. The first region, comprised of 164 ORFs, shows a complex mosaic organization, incorporating genes acquired from diverse sources like ISs, composite Tns, plasmids, and phages [60]. Crucially, the second region, comprising 113 ORFs (ORF165 to ORF278), is a complete copy of the PAGI-2. The presence of various Tnps, specifically those belonging to the IS3 (IS*Psy37* and IS*222*) and IS*110* families, has been confirmed in the first, mosaic region of PAGI-47 [60]. Some ORFs encode TraG and TraD proteins, which contribute to the conjugation and integration system, and some ORFs encode hypothetical proteins [60,256].

### 7.17. PAGI-48’s Structure and Functional Characteristics

PAGI-48, with a similarity of 48% with PAGI-8, is built of eight ORFs. In this regard, ORFs 1, 2, and 4 are shared between PAGI-48 and PAGI-8. A phage-associated Intr, a hypothetical protein, and a TraY/DotA family-related conjugal transfer protein are encoded by these common ORFs, respectively. A recombination protein has been detected, which is encoded by ORF3 [60].

### 7.18. PAGI-49’s Structure and Functional Characteristics

PAGI-49, a 19-ORF-structure, is capable of encoding a variety of functions. Integration into the host genome is likely mediated by the phage Intr encoded by ORF2. ORFs 3 and 4 encode RHS family proteins, related to polymorphic toxins in bacterial exotoxins. The aminoglycosides resistance feature has been identified by expression of the GNAT family N-acetyltransferase, encoded by ORF8. Virulence-associated protein E (VapE), is encoded by ORF13. Finally, an Alpha family transcriptional regulator is encoded by ORF19. The rest of them (13 ORFs) encode hypothetical proteins [60,257,258].

### 7.19. PAGI-50’s Structure and Functional Characteristics

The PAGI-50 region encompasses 56 ORFs, but a slight majority (52%) are predicted to encode hypothetical proteins. Notably, the region also contains a multitude of *tnp* genes belonging to diverse IS families, including IS*Pa57*, IS*Pa37*, IS*222*, IS*Pa47*, IS*Sal1*, IS*Pa8*, and IS*1474* [60].

### 7.20. PAGI-51’s Structure and Functional Characteristics

Although PAGI-51 shares 64% sequence identity with PAGI-50, with high similarity at both the 5′ and 3′ ends (including the first two and last 27 ORFs), some functional differences exist. PAGI-51 harbors a distinct ABC-type transport system, composed of ORFs 10–23, which is not present in PAGI-50. This transport system allows the bacteria carrying PAGI-51 to detoxify sulfur compounds [60].

### 7.21. PAGI-52’s and PAGI-553’s Structures and Functional Characteristics

PAGI-52 possesses 61 ORFs. These ORFs include a type II TAS, ARGs (e.g., *bla*_GES-1_, *ant(2′), aac(6′)-II*, streptomycin adenyltransferase, *bla*_OXA-2_, *qacE delta1* and *sul1*), HMRGs (e.g., *mer* operon genes; mercury resistance genes) and mobility-related genes. The presence of Tns such as Tn*As3* and ISs like IS*1326*, IS*Pa1635*, significantly enhances the plasticity and genetic exchanges of PAGI-52. PAGI-53 is built of 52 ORFs and may play as a metabolic island. ORFs 1 and 2 and ORFs 4–7 encode two type II TAS modules and an ABC-type transport system [60].

## 8. Conclusions

As aforementioned, *P. aeruginosa* possesses a mega-pan-genome with a high genomic versatility and plasticity, which makes it a super-powerful bacterium to survive in different environmental conditions with a variety of mechanisms and strategies. On the other hand, there is a wide range of known and unknown GEs, and in particular TEs and jumping genes, which facilitate the transmission of different ARGs from a bacterial cell to others. As we discussed within the current literature, together with ARGs, a wide range of VGs, HMRGs, and MGs can be transferred and disseminated. This feature makes the antimicrobial therapeutic strategy for the infections caused by *P. aeruginosa* bacterial strains more difficult and complicated. A versatile range of gene cassettes, genetic platforms, and resistance genes represents an uncertain future relating to the use of antimicrobial therapy against bacterial infections comprising CRPA. Thus, we believe that other new strategies should be considered in this regard. In brief, these series of studies and investigations including our literature review have some certain limitations including a wide range of known and unknown GEs, a wide range of known and unknown environmental factors, a wide range of known and unknown mutations and SNPs, a wide range of known and unknown genetic exchanges, a wide range of known and unknown genomic characteristics relating to different organisms, a wide range of known and unknown factors which may lead to appearance of a wide range of synergistic or antagonistic effects in molecular and genetic levels. All of these items are the main determinants of the appearance and development of different types of resistance genes, etc. Thus, this field of science is a great challenge for the scientists; a puzzle with a wide range of present and absent puzzle pieces!

In recent years, a noteworthy tool named “Artificial Intelligence” (AI) has been introduced [259,260]. Is it possible to recruit the AI approach for overcoming this huge challenge in the future? Is it possible to employ this new technology to disarm the resistome and the virulome of the superbugs? Is it possible to utilize this new tool to produce new effective antibiotics? At this moment, nobody knows. Time will answer these questions.

## Figures and Tables

**Table 1:** General characteristics of genomic islands (GIs) in *P. aeruginosa*.

Pathogen	Genomic Islands	Size	GC	Insertion Site	References
*Pseudomonas aeruginosa*	*P. aeruginosa* genomic island 1 (PAGI-1)	~49 (48.9) kbp	first 75% of the sequence: 63.7% remaining 25% of the sequence: 54.9%	-	[48,49,50]
	PAGI-2 (hypervariable region, with a bipartite structure)	~110 kbp (105 kbp + ~7 kbp)	64.7% + 66.1%	tRNA^Gly^	[49]
	PAGI-3 (hypervariable region, with a bipartite structure)	~110 kbp (103 kbp + ~7 kbp)	59.2% + 66.1%	tRNA^Gly^	
	PAGI-4	23.4 kbp	56.0%	tRNA^Lys^	[51]
	PAGI-5	99 kbp	59.6%	tRNA^Lys^	[52,53]
	PAGI-6	44.3 kbp	60.8%	tRNA^Thr^	[50]
	PAGI-7	~22.5 kbp	55.8%	-	
	PAGI-8	~16.2 kbp	54.1%	tRNA^Phe^	
	PAGI-9	~6.6 kbp	63.4%	-	
	PAGI-10	~2.2 kbp	56.6%	-	
	PAGI-11	~2.0 kbp	50.5%	-	
	PAGI-12	124 kbp	64.3%	tRNA^Lys^	[54]
	PAGI-13	~197.4 kbp	62.3%	tRNA^Gly^	[55]
	PAGI-14	~47.5 kbp	55%	tRNA^Pro^	
	PAGI-15	~118.7 kbp	61.3%	tRNA^Gly^	[56]
	PAGI-16	~95 kbp	61.4%	tRNA^Gly^	
	PAGI-17	~117 kbp	64.1%	tRNA^Gly^	[57]
	PAGI-18	~21 kbp	-	tRNA^Gly^	[58]
	PAGI-25	~103.3 kbp	-	tRNA^Gly^	[59]
	PAGI-41	~184.0 kbp	59.43%	-	[60,61]
	PAGI-42	~188.4 kbp	59.36%	-	
	PAGI-43	318.5 kbp	59.0%	-	
	PAGI-44	~135.5 kbp	58.6%	-	[60,62]
	PAGI-45	~58.94 kbp	60.5%	-	
	PAGI-46	~96.8 kbp	62.8%	tRNA^Gly^	[60,61,62]
	PAGI-47	~252.8 kbp	63.1%	tRNA^Gly^	
	PAGI-48	~13.9 kbp	54.21%	tRNA^Phe^	[60,61]
	PAGI-49	~15.8 kbp	61.5%	tRNA^Phe^	[60,62]
	PAGI-50	~75.4 kbp	57.48%	tRNA^Arg^	[60,61]
	PAGI-51	~93.7 kbp	59.67%	tRNA^Arg^	
	PAGI-51	~93.5 kbp	59.66%	tRNA^Arg^	
	PAGI-52	~55.5 kbp	58.31%	-	
	PAGI-53	61.7 kbp	55.65%	-	[60,62]

## Data Availability

No new data were created or analyzed in this study.
